# Wetland Suitability and Connectivity for Trans-Saharan Migratory Waterbirds

**DOI:** 10.1371/journal.pone.0135445

**Published:** 2015-08-10

**Authors:** Ronny Merken, Evelien Deboelpaep, Joachim Teunen, Santiago Saura, Nico Koedam

**Affiliations:** 1 Laboratory of Plant Science and Nature Management, Vrije Universiteit Brussel, Brussels, Belgium; 2 Department of Natural Systems and Resources, Polytechnic University of Madrid, Ciudad Universitaria s/n, Madrid, Spain; Università degli Studi di Milano-Bicocca, ITALY

## Abstract

To complete their life cycle waterbirds rely on patchily distributed and often ephemeral wetlands along their migration route in a vast unsuitable matrix. However, further loss and degradation of remaining wetland habitats might lead to a configuration and size of stopovers that is no longer sufficient to ensure long-term survival of waterbird populations. By identifying optimal conservation targets to maintain overall habitat availability *en route*, we can accommodate an as yet absent functional connectivity component in larger management frameworks for migratory waterbirds, such as the Ramsar Convention and the EU Natura 2000 Network. Using a graph-based habitat availability metric (Equivalent Connected Area) we determine the functional connectivity of wetland networks for seven migratory waterbirds with divergent habitat requirements. Analyses are performed at two spatial extents both spanning the Mediterranean Sea and centered around Greece (Balkan-Cyrenaica and Greece-Cyrenaica). We create species-specific suitable habitat maps and account for human disturbance by species-specific disturbance buffers, based on expert estimates of Flight Initiation Distances. At both spatial extents we quantitatively determine the habitat networks’ overall functional connectivity and identify wetland sites that are crucial for maintaining a well-connected network. We show that the wetland networks for both spatial extents are relatively well connected and identify several wetland sites in Greece and Libya as important for maintaining connectivity. The application of disturbance buffers results in wetland site-specific reduction of suitable habitat area (0.90–7.36%) and an overall decrease of the network’s connectivity (0.65–6.82%). In addition, we show that the habitat networks of a limited set of species can be combined into a single network which accounts for their autoecological requirements. We conclude that targeted management in few but specific wetland complexes could benefit migratory waterbird populations. Deterioration of these vital wetland sites in Greece and Libya will have disproportionate consequences to the waterbird populations they support.

## Introduction

Equipped with sublime navigating and flight abilities, long-distance migratory waterbirds make impressive seasonal movements from breeding grounds to wintering areas and back to complete the annual cycle [1, 2]. Since waterbirds often are unable to travel between wintering and breeding grounds in a single flight, they must use stopovers [3]. These wetland stepping stones are patchily distributed and often ephemeral in a human-dominated unsuitable matrix [1]. The cumulative amount of time spent at stopover sites, which far exceeds time spent in flight, accounts for the greatest part of the total migration period [4]. Particularly high mortality rates which can be up to six times higher during migration than during stationary periods reflect the substantial risks waterbirds are confronted with *en route* [1, 5].

Phenotypic and evolutionary plasticity of migration patterns, schedules and strategies allow birds to adapt to (abrupt) environmental changes. However 40% of more than 100 Afro-Palearctic migrant species show severe and sustained declines [6, 7]. Since (long-distance) migratory birds integrate the state of ecosystems at large temporal and spatial scales, they are regarded as sentinels of the general state of the environment [8]. Their decline may thus be a warning signal for environmental degradation.

Especially loss and degradation of wetland stopovers are impacting waterbird populations by lowering migratory performance and even survival or reproduction [1]. Wetlands are among the most threatened habitats worldwide, with approximately half of all wetlands which existed in the last century have now been lost due to agriculture, urban expansion and management practices [9, 10]. Specifically for Greece, more than 60 percent of all wetlands have been lost between 1950 and 1985 [10].

Wetland habitat suitability describes the relation between a species’ habitat requirements, i.e. the fundamental niche, and wetland habitat factors affecting use of a site [11, 12]. When interpreting habitat suitability models, we are confronted with the realized niche where biotic interactions and the species’ dispersal abilities come into play [13] and habitat selection, which is inherently highly scale-sensitive, both temporally and spatially [14]. Even though the total wetland size can determine waterbird diversity, the amount of suitable habitat is more important to waterbirds since not all wetland biotopes are suitable with regard to the birds’ morphology or ecological habits [15].

Habitat availability is an important concept for migratory birds since it integrates habitat patch area (or other patch attributes like habitat quality or suitability) and interpatch connectivity [16]. Ecological connectivity (not to be confused with migratory connectivity [17]), has a long-standing research history [18] but applying it to migration is not straightforward. Migratory movements involve a complex set of behavioural decisions based on the condition of the individual bird (such as mass and fat percentage), the availability and quality of stopover habitats, and their spatial array along the migration route [2, 19]. How migratory movements are affected by these factors is poorly known [20].

In theory, in the process of wetland habitat reduction, an absolute minimum in configuration and size of stopovers needs to be preserved in order to ensure long-term survival of waterbird species across their ranges [21]. Quantitative criteria assessing the relative importance of wetland sites (e.g. number of individuals or proportion of the individuals in a population) are a straightforward and justifiable tool for the identification of wetlands of international importance. The true conservation significance of particular sites within a migratory network might however be missed [22] considering the complexity of migratory patterns, dynamicity of wetlands and climate change. The latter has the potential to negatively impact the ecological integrity of ecosystems—i.a. due to prolonged droughts in wetlands—in the Mediterranean and Sahel regions, thereby aggravating the migratory challenge waterbirds are confronted with [23, 24].

Various modelling approaches considering stopover habitats along migration routes [3, 25, 26] allowed us to gain initial insight into potential ecological connectivity for migratory birds, with suggestions for reserve design. In particular graph-theoretic models, which include spatially explicit habitat data and species-specific dispersal data, are a very effective and efficient approach to provide a reasonably detailed picture of potential connectivity combined with modest data requirements [27].

In this study, we used a graph-based habitat availability metric (the Equivalent Connected Area corresponding to the Probability of Connectivity (ECA(PC), [16]) during a spring migration period of seven trans-Saharan migratory waterbirds with divergent habitat requirements to quantitatively assess the overall functional connectivity and to identify important sites to uphold connectivity of the network. Analyses were performed at two spatial extents (Balkan-Cyrenaica and Greece-Cyrenaica) in the eastern part of the Mediterranean Region which provides crucial stopover sites to overcome the particular geographic configuration of sizeable barriers nearby (the Sahara desert, the Mediterranean Sea and several mountain ranges such as the Alps, the Balkans and Carpathian mountain ranges). Human activities, modified landscapes and successive jurisdictions with respective policies further define the features of the environment of migratory waterbirds. The strong nature and biodiversity policy which is enforced by the European Union, with the ‘Natura 2000’ network as its tangible outcome, is partly based on transboundary ecological considerations and international cooperation for wildlife conservation. It only applies to part of our study area (i.e. EU member states Slovenia, Croatia and Greece), which can entail a legislative and management mismatch and international conservation incoherence with non-EU member states. This may affect the integrated quality of the migratory route.

Since many economic and recreational activities in the Mediterranean are situated in or around wetlands [28], we incorporated the often overlooked impact of human presence in stopover habitats by estimating species-specific Flight Initiation Distances (FID) at which the animal is expected to flush or move away from the approaching disturbance source [29]. This parameter represents one of the many sources of human disturbances affecting waterbirds by constraining the availability of suitable habitat. FID is a parameter which is relatively easy to quantify. It is considered to be a conservative basis for developing buffers that could alleviate the harmful effects of human disturbance to waterbirds [29, 30]. Buffer distances were determined on basis of an expert survey yielding expert estimates for FIDs of six waterbird species (Table 1).

**Table 1:** Selection of suitable habitat categories at both spatial extents.

Extent	Species	CORINE Land Cover codes (CLC2006)													
		2.4.3.	3.3.1.	4.1.1.	4.2.1.	4.2.2.	4.2.3.	5.1.1.	f5.1.2.	s5.1.2.	5.2.1.	5.2.2.	Reed beds	Artificial lakes	FID (m)
**Balkan- Cyrenaica**	/			x	x	x	x		x	x	x				/
**Greece-Cyrenaica**	*Ardea purpurea*												x		55
	*Ardeola ralloides*			x				x*	x*				x	x	25
	*Calidris ferruginea*		x		x	x	x			x*					30
	*Chlidonias niger*		x	x	x	x	x	X	x	X	x	X	x	x	/
	*Egretta garzetta*		x	x	x	x	x	x*	x*	x*	x*	x*		x	35
	*Himantopus himantopus*			x	x		x		x*	x*	x*	x*			30
	*Tringa glareola*	x		x			x	x*	x*					x	35

**Balkan-Cyrenaica**: Choice of wetland habitat categories from existing CLC2006 data. Categories comprise: Inland marshes (4.1.1.), Coastal & salt marshes (4.2.1. / 4.2.2. / 4.2.3), Inland water bodies (5.1.2.) and Inland & coastal waters (5.1.2. / 5.2.1.). **Greece-Cyrenaica**: Choice of habitat categories following CLC2006 used for the species-specific analyses with a manual interpretation of the satellite images: ‘2.4.3.’ (agricultural land with significant areas of natural vegetation), ‘3.3.1.’ (beaches, dunes and sands), ‘5.1.1.’ (water channels) and ‘5.2.2.’ (estuaries). 'f5.1.2.' and 's5.1.2.' stand for water bodies with fresh and salt water respectively. ‘X’-es indicate habitat types considered to be suitable for a particular species. When an '*' is indicated for a ‘water category’ (5.x.x.), only a water buffer of 0.30 m from polygon edges was included in the analysis as a hypothetical wading habitat for waterbirds. The Flight Initiation Distance buffer (m) (or disturbance distance) is also shown.

By identifying optimal targets for conservation to maintain the overall habitat availability in the area, we can accommodate an as yet absent functional connectivity component into larger planning and management frameworks for migratory waterbird species.

## Materials and Methods

### Spatial extents

**Fig 1:**
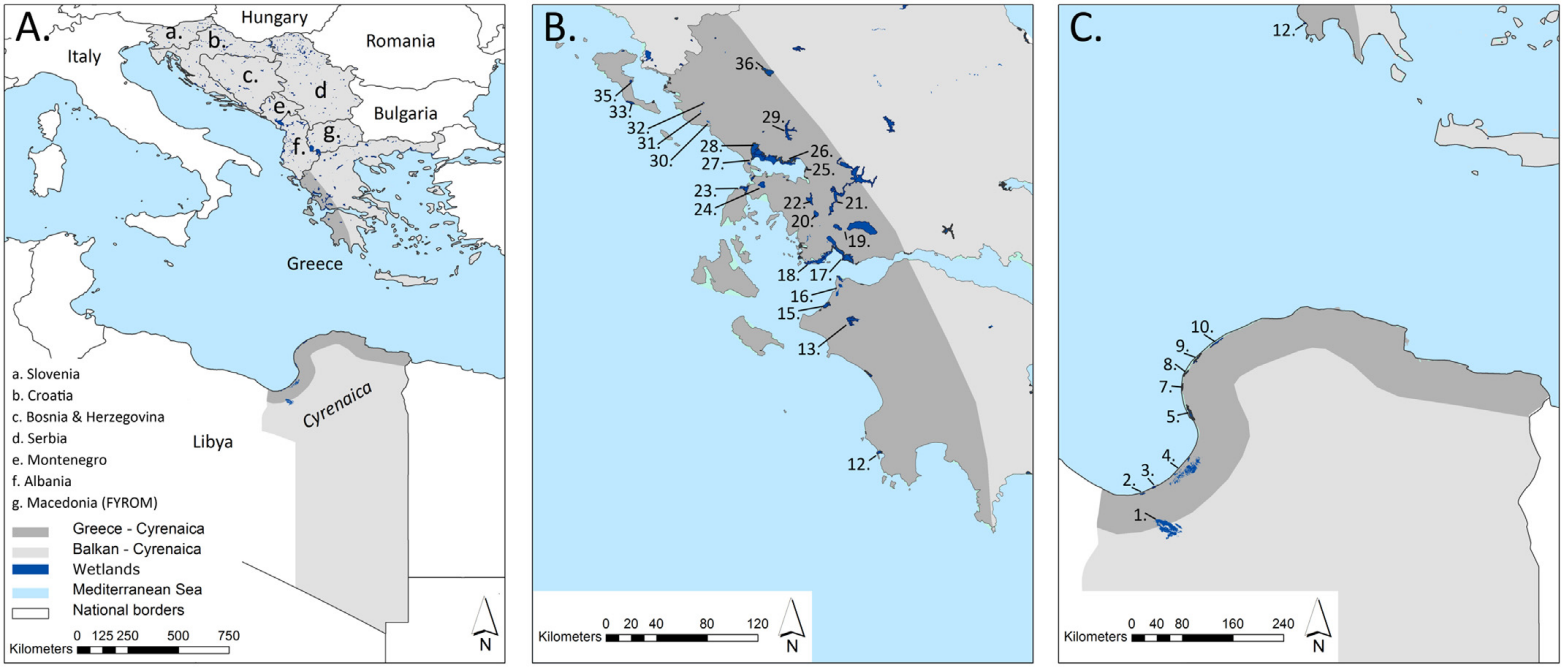
Delineation of the study area with indication of wetlands. **A** Balkan-Cyrenaica: the number of patches for different wetland habitats at this extent can be consulted in Table 2; **B** and **C** Greece-Cyrenaica encompasses a 65 km coastal band in Greece (**B**) and Cyrenaica (Libya) (**C**). Focal wetlands are: (1) Sebkha Al Kabirah; (2) Sebkha Bishr; (3) Sebkha Hafiroun & Brega; (4) Sebkha Ajdabiya & Al Brayqah; (5, 6) Sekbha Karkurah; (7) Ghemines; (8) Sebkha Gandoufa; (9) Sebkha El Thama & Esselawi; (10, 11) Sebkha Al Kuz; (12) Pylos; (13) Techniti Limni Pineiou; (15,16) Strofilia, Kotychi & Araxos; (17, 18) Messolonghi; (19) Trichonis & Lysimacheia; (20) Ozeros; (21) Techniti Limni Kastrakiou-Stratiou; (22) Amvrakia; (23) Lefkada; (24) Voulkaria; (25,26,27,28) Amvrakikos; (29) Techniti Limni Pournariou; (30) Acheron; (31) Kalodiki; (32) Kalodiki North; (33) Korissia; (34) Kalamas; (35) Chalikiopoulou; (36) Ioannina. Neither Kosovo nor Cyrenaica were taken up as separate states in our text or maps, a practical issue which does not reflect a political judgement. See [34] and [33] for datasets of national borders.

Given the scale-dependency of connectivity we worked at two different spatial extents which both span the Mediterranean Sea. We selected a broad scale region (Balkan-Cyrenaica) where we included wetland habitats of eastern Libya (Cyrenaica) and of most countries of the Balkan Peninsula (excluding Bulgaria, Romania and Eastern Thrace (Turkey), Fig 1A). Furthermore, in a nested fine scale region (Greece-Cyrenaica) we considered wetlands of the western Greek mainland, the Ionian Islands and eastern Libya (Cyrenaica). For this spatial extent, we only included wetlands—either as single systems or as wetland complexes—situated (a) within a 65 km coastal band and (b) larger than 500 hectares (Fig 1A and 1C).

The justification for this coastal band (a) along the Greek and Libyan coast is based on the predictability of resource availability and hydrological stability of coastal wetlands to migratory birds, as opposed to inland wetlands [31]. For Greece, this 65 km band follows the north-south orographical barrier presented by the Pindos (Greek mainland) and Taygetos (Peloponnese peninsula) mountain ranges parallel to the coast. Migrating waterbirds often avoid crossing high mountains and other landscape elements that pose an increased (energetic) challenge to these birds. Hence, they are funnelled along mountain ranges, sea-coasts and other ‘leading lines’ [1]. We applied this 65 km coastal band to Libya for consistency, which is appropriate as well since there are no large (>500 ha) inland wetlands with large stretches of suitable habitat outside this 65 km limit.

The size criterion of 500 ha (b) is based on the assumption that larger wetlands serve as beacons for migratory birds *en route*, which are attracted to water surfaces reflecting solar radiation [32, 33]. Larger wetlands have a higher probability to comprise different habitat types, thus having higher habitat heterogeneity and waterbird species diversity [12]. It is important to distinguish between the 500 ha size selection criterion and the actual size of habitat patches for one wetland complex, as they were taken up in the connectivity analysis (further described in ‘Data sources and wetland classification’). Habitat patch (node) size for a given species might range from very small (e.g. 19 m^2^ at Ghemines for *Tringa glareola*, Fig 1C) to very large (e.g. 1279 km^2^ at Sebkha Al Kabirah for *Egretta garzetta*, Fig 1C).

### Selection of bird species

We selected seven wetland-dependent species (Table 1; S1 File) with divergent habitat requirements to construct an ‘ecological umbrella’ that integrates different feeding and habitat selection strategies. This selection includes both stenotopic and eurytopic species, which respectively have restricted and broad ecological niches[35]. As such they are representative of a much wider diversity of waterbird species. All species are trans-Saharan migrants and frequent Greece at least in moderate numbers. Except for *Calidris ferruginea* all species require the designation of Special Protection Areas (SPAs) within the European Union since they are vulnerable to specific changes in their habitat and are hence listed on Annex I of the EU’s Birds Directive (2009/147/EC). All focal species are however quite common and are labelled to be of ‘Least Concern’ globally according to the International Union for Conservation of Nature (IUCN) criteria.

### Data sources and wetland classification

For Greece-Cyrenaica we downloaded recent high-resolution satellite images of 2013 and 2014 preferably of the spring season from Google Earth Pro. We focused on spring migration since this phase is more time-limited for birds than autumn migration [36]. When no suitable recent satellite images from spring were available, we selected the most recent images from summer or autumn, that were however representative for the status of wetlands during the spring migration phase. In this way we could account for some important changes that occurred in recent years for some wetlands. Since the hydrological regime of wetlands is dynamic within and across annual cycles, the temporal window of selected images directs the status and accessibility of certain (feeding) habitats [12]. Therefore, we selected images that were representative for the status of wetlands during the spring migration phase.

**Fig 2:**
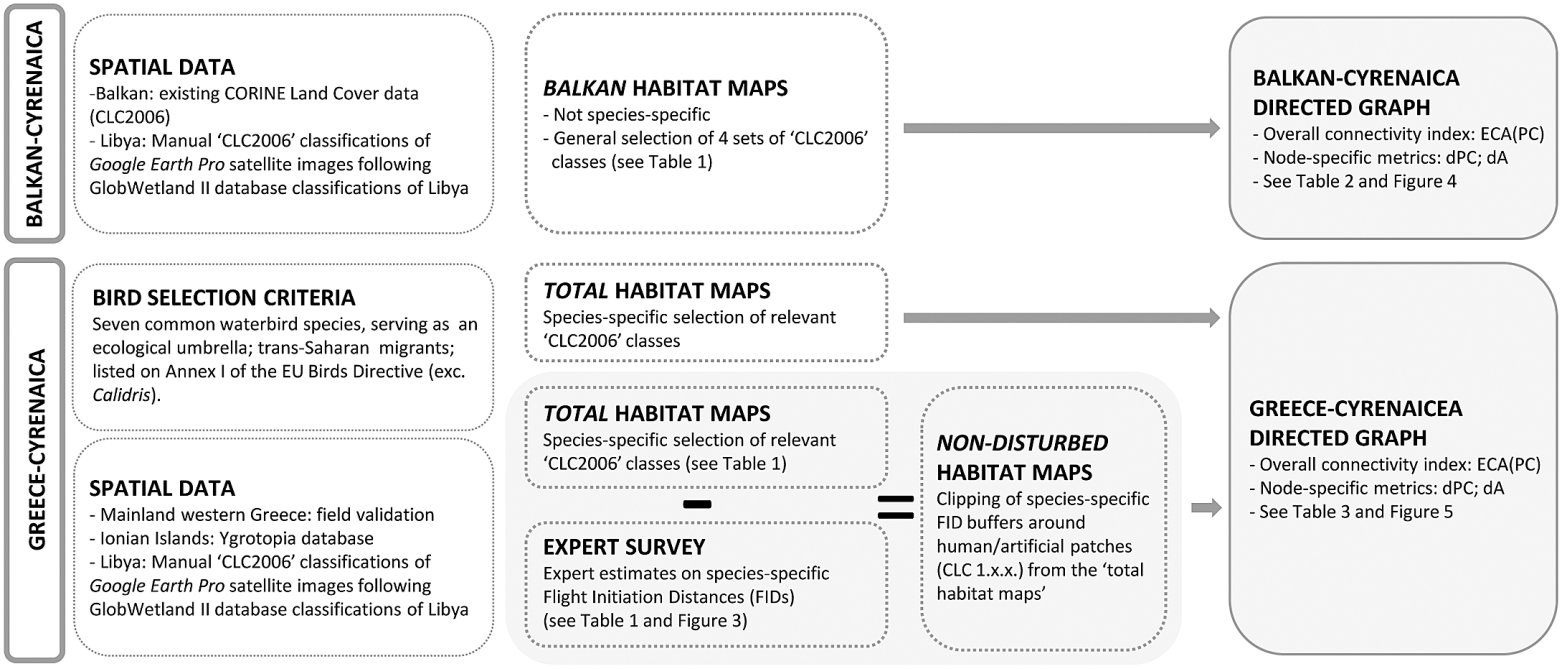
Workflow of the analysis indicating the different input data, approaches and output.

Satellite images were imported and georeferenced in ArcGIS, and used along ground truth data to meticulously delineate and classify wetland habitat patches manually according to CLC2006 classes. We always merged all habitat polygons less than 5 km apart into larger habitat patches (nodes), since this distance is easily travelled by the selected bird species. Otherwise, without this merging operation, the relevance of identifying important wetland sites would have been impaired. Multiple ID-codes were assigned to a single wetland complex when distinct clusters of suitable habitat were farther apart than 5 km (e.g. for Amvrakikos: 25 = south, 26 = east, 27 = west, 28 = north; Fig 1). In April and May 2012 we performed extensive field validations in all focal wetlands of the western Greek mainland. For wetlands of the Ionian Islands, we accessed the Oikoskopio and Ygrotopio Islands databases, developed by WWF Greece, as additional baselines (respectively available from [37] and [38]; Fig 2).

For Balkan-Cyrenaica we used existing CORINE Land Cover data (CLC2006), which are freely available online for the EU territory [39]. We selected four different sets of CLC classes (Table 1 and Fig 2) representing the most important categories for our focal species [35].

The same Cyrenaican wetlands are used for both spatial extents since there were no other large Cyrenaican wetlands outside this 65 km coastal band: the hyper-arid Sahara desert already reaches the coast in eastern Cyrenaica while the west only has a narrow strip of land with a Mediterranean climate. We relied on the free online available GlobWetland II WebGIS-tool of the European Space Agency and Ramsar secretariat for our Libyan classifications (1:50.000 scale; [40]), because wetlands of Libya were inaccessible for ground truthing due to the insecurity issues in the region.

### Habitat maps

Based on the species’ autoecological requirements [35] we selected all relevant CLC2006 categories constituting suitable habitat patches per species for the Greece-Cyrenaica analysis (Table 1). We incorporated wetland size, vegetation and salinity, but data on food availability for all wetlands at this spatial extent are not available. Species-specific habitat suitability maps were created in ArcGIS 10 (ESRI). Additionally, we created open water body margins of 0.30 m from polygon edges (all polygons labelled 5.x.x.; indicated with an 'X' in Table 1) since wetland-specific topography and water level dynamics were unavailable for most wetland sites. We considered this minimal buffer to be relatively shallow and its application should be regarded as an intermediate and conservative solution to be able to include existing suitable habitat along the great diversity of water body margins for the selected bird species. We conjectured that the inclusion of this water body margin would be far better than assuming there would not be any suitable habitat along the water bodies for wading waterbirds, even though longer legged species might access deeper waters than the short legged [2].

### Expert survey

In order to obtain species-specific expert estimates for Flight Initiation Distances (FIDs), we conducted an expert survey from August 2013 until February 2014. We collected author names and names from the acknowledgements from published articles in which (one of) our species appear(s), thereby including experts that have actively worked with the species in the field. Additionally, we asked to forward our survey to knowledgeable people from among their acquaintances. Up to three reminders were sent to every respondent. In total, 839 emails were sent to 387 experts. Kruskall-Wallis tests for differences in species-specific FID results and post-hoc pairwise comparisons by means of the Mann Whitney U statistic were performed in Statistica (version 8.0). This analysis forms the basis for the species-specific disturbance buffer, for which we must identify different patterns of the focal species’ response to human disturbance. See S1 File for the expert survey form.

### Non-disturbed habitat maps

Based on the expert estimates for species-specific FID, we derived a conservative disturbance distance guideline by rounding the estimates’ average to the nearest quintuple (Table 1). Around every artificial patch, we created a disturbance zone, which was clipped from the habitat maps. The landscape elements for which we applied such a disturbance buffer (CLC 1.x.x., S2 File) include all buildings, transportation networks (asphalted as well as gravel roads), (air)ports and leisure sites where we expect human presence to be prominent.

As a result, we obtained the non-disturbed habitat map with the remaining ‘suitable’ habitat area. For every species, parallel analyses were executed with the habitat maps and non-disturbed habitat maps. The latter does not apply to *Chlidonias niger*; while migrating this species is less sensitive to disturbance (compared to the other focal species) given its constant foraging flight. It mostly experiences negative effects of human presence while breeding [35, 41] (Table 2).

**Table 2:** The total surface area and Equivalent Connected Area ECA(PC) for Balkan-Cyrenaica for a directed graph.

	Total surface area (km^2^)	ECA_min_ (km^2^)	ECA (km^2^)
Coastal marshes, salinas and intertidal flats 4.2.X. (79 nodes)	557.55	146.09	375.24
Inland marshes 4.1.1. (253 nodes)	1,710.25	581.01	1,120.35
Water bodies 5.1.2. (397 nodes)	2,839.16	648.12	1,924.41
Water bodies and coastal lagoons 5.1.2./5.2.1. (410 nodes)	3,171.79	660.20	2,147.68

### Modelling

We used a graph-based habitat availability metric that quantifies functional connectivity: the Probability of Connectivity index (PC) [16, 42]. It is defined as the probability that two animals randomly placed within the landscape fall into habitat areas that are reachable from each other (interconnected), given a set of habitat patches and the connections (p_ij_) among them. The standard way the PC index has been applied considers undirected or symmetric ‘dispersal’ probabilities (the probability of dispersal from patch i to j being the same as from patch j to i). However, for calculating a migratory connectivity between wetland patches in spring we need a directed or asymmetrical graph (*i*.*e*. a graph in which the probability of movement (dispersal) from i to j (p_ij_) is different from the probability of movement from j to i (p_ji_) for pairs of patches ij). We used a purposefully adapted Conefor version for directed graphs [43, 44] to implement directed dispersal probabilities for Balkan-Cyrenaica and Greece-Cyrenaica. Wetlands were represented in the network’s different nodes through the surface areas of their constituting habitat patches, while all pairwise node-to-node distances or probabilities were considered as connections between the nodes.

Surface area calculations of merged habitat patches per species were performed using the Mollweide equal area projected coordinate system in ArcGis (in our case in square meters) and interpatch distances (based on feature spherical centroids) were calculated using the world Azimuthal Equidistant projection.

Conefor further requires setting a distance value along with a corresponding probability to convert all interpatch distances into interpatch dispersal probabilities by means of a decreasing exponential function used for the computation of the PC index. We modelled the maximum flight distance of *Ardea purpurea* and *Calidris ferruginea* (with contrasting wing span and area) at three different energetic conditions (fat percentages of 15%, 25% and 35%) using the software Flight v1.24 (C. Pennycuick; Bristol University) to attain a representative and realistic threshold distance with corresponding probability for these model species (data not shown). The parameter input in Conefor was set using a maximum distance for a non-stop flight of 8,000 km, corresponding to a probability of 0.05. We constructed the direct probability input files by using the interpatch dispersal probabilities which were automatically generated by Conefor. We provided all northbound, northeast-bound and northwest-bound paths (300°-60° azimuth) with the calculated interpatch dispersal probabilities of Conefor while all southward paths (120°-240° azimuth) were given a probability of ‘0’. The remaining azimuths did not occur in our system.

The output of the models consists of two parts: the overall connectivity index 'Equivalent Connected Area' or ECA(PC) [45]; and the node-specific metrics dA (the percentage of total habitat area represented by a single node), and dPC (the relative importance of a single node). ECA(PC) is defined as the size of a single habitat patch (maximally connected) that would provide the same value of the Probability of Connectivity (PC) than the actual habitat pattern in the landscape. In case of a maximally connected network, ECA(PC) is equal to the total habitat area of the network (upper limit of ECA(PC)). The minimal ECA(PC) value ECA_min_ is given by eq (1), with a_i_ the attribute (i.e. surface area) of node i and k the number of nodes. ECA_min_ corresponds to a minimally connected network [46].

$$ECA_{min}\;=\;\sqrt{\sum_{i=1}^{k}a_{i}^{2}}$$ (1)

Node importance values dPC are partitioned into dPC*intra*, dPC*flux* and dPC*connector*, representing the different fractions in which patches and links contribute to habitat availability within the network. The first fraction, dPC*intra*, an intrapatch metric, corresponds to the habitat area of a focal patch. The second, dPC*flux*, is the dispersal flux to and from a particular patch, depending on its position in the network, weighed by the habitat area of that patch. dPC*connector* accounts for the importance of a patch as a stepping stone in between other habitat patches, independent of its surface area. Higher dPC values are assigned to habitat patches that more profoundly contribute to maintaining overall connectivity [16, 47].

Additionally, in order to summarise the importance of each node targeted to groups of species instead of single species, we calculated the mean dPC value for each node for all species for which that node was present in the habitat network.

## Results

### Expert survey

The survey was answered by 104 experts whereas 29 respondents provided expert estimates. In sum, 167 Flight Initiation Distance (FID) expert estimates were received.

**Fig 3:**
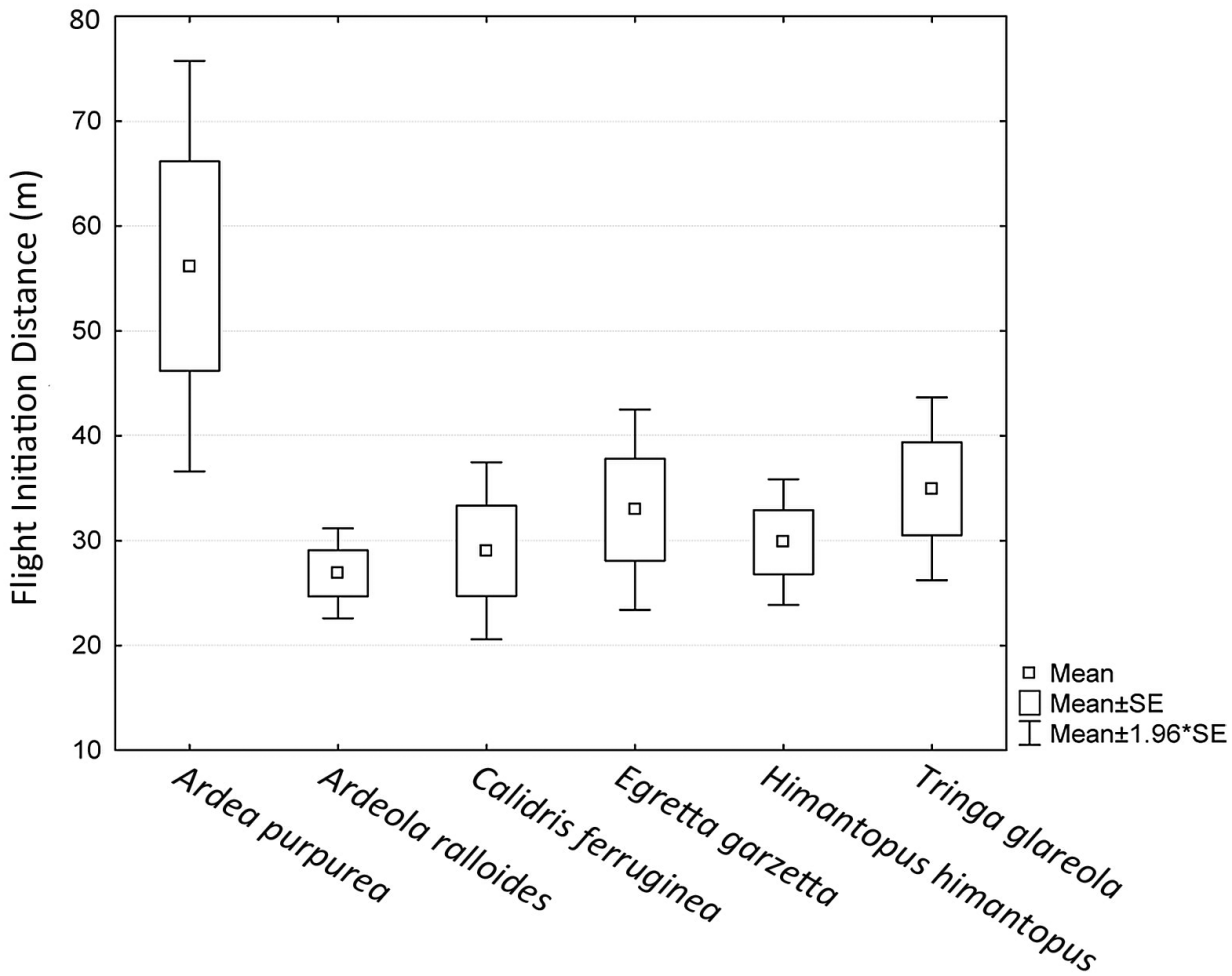
Expert estimates of the Flight Initiation Distance (FID).

Kruskall-Wallis H tests were not significant (0.05 level) for differences among species for FID results (H(5) = 10.22381, p = 0.0691, N = 109). Post-hoc pairwise comparisons by means of the Mann Whitney U statistic revealed a tendency of *Ardea purpurea* disturbance distances to be different from the other species. These differences were significant for the comparisons with *Ardeola ralloides* (U = 55, N1 = N2 = 17, p = 0.002), *Calidris ferruginea* (U = 88, N1 = 17, N2 = 18, p = 0.03); *Egretta garzetta* (U = 107, N1 = 17, N2 = 21, p = 0.04) and *Himantopus himantopus* (U = 79, N1 = 17, N2 = 18, p = 0.01). This pattern is also reflected in the box-and-whisker plots (Fig 3). Hence, our results indicate that there is some variation in disturbance distances among species and that only *A*. *purpurea* shows a divergent pattern for FID (Table 2).

### Habitat reduction

For most species the habitat reduction due to disturbance varies around 2 percent (Table 3), excepting *A*. *ralloides* where the difference between habitat and non-disturbed habitat maps is smallest, and *A*. *purpurea* which is most affected in terms of surface area reduction given its small amount of suitable habitat. The observed variations are consistent with our expectations based on the respective buffer distances that were applied.

**Table 3:** Results of the Greece-Cyrenaica directed graph analysis.

Species	Habitat maps	Total surface area (km^2^)	ECAmin (km^2^)	ECA(PC) (km^2^)
*A*. *purpurea*	Total	58.65	36.10	47.81
	Non-disturbed	54.33	34.32	44.55
	*Difference (%)*	*7*.*36*	*4*.*95*	*6*.*82*
*A*. *ralloides*	Total	875.94	551.40	712.64
	Non-disturbed	868.02	549.51	708.07
	*Difference (%)*	*0*.*90*	*0*.*35*	*0*.*64*
*C*. *ferruginea*	Total	810.34	537.37	668.71
	Non-disturbed	788.98	531.75	655.25
	*Difference (%)*	*2*.*64*	*1*.*04*	*2*.*01*
*C*. *niger*	Total	3,373.80	1,639.67	2,500.09
	Non-disturbed	-	-	-
	*Difference (%)*	*-*	*-*	*-*
*E*. *garzetta*	Total	2,479.61	1,529.77	2,006.26
	Non-disturbed	2,417.66	1,521.17	1,983.97
	*Difference (%)*	*2*.*50*	*0*.*56*	*1*.*11*
*H*. *himantopus*	Total	968.59	554.19	763.76
	Non-disturbed	950.72	551.50	753.49
	*Difference (%)*	*1*.*85*	*0*.*48*	*1*.*35*
*T*. *glareola*	Total	853.06	550.29	701.58
	Non-disturbed	839.71	547.67	694.15
	*Difference (%)*	*1*.*56*	*0*.*48*	*1*.*06*

A comparison of total habitat (Total) and total non-disturbed habitats (Non-disturbed) maps is made by means of total habitat surface, ECA_min_ and Equivalent Connected Area through the Probability of Connectivity (ECA(PC)) values, with indication of differences in percentages. Since an FID distance for *C*. *niger* was less relevant, no disturbed habitat area was established.

### Wetland connectivity in Balkan-Cyrenaica

**Fig 4:**
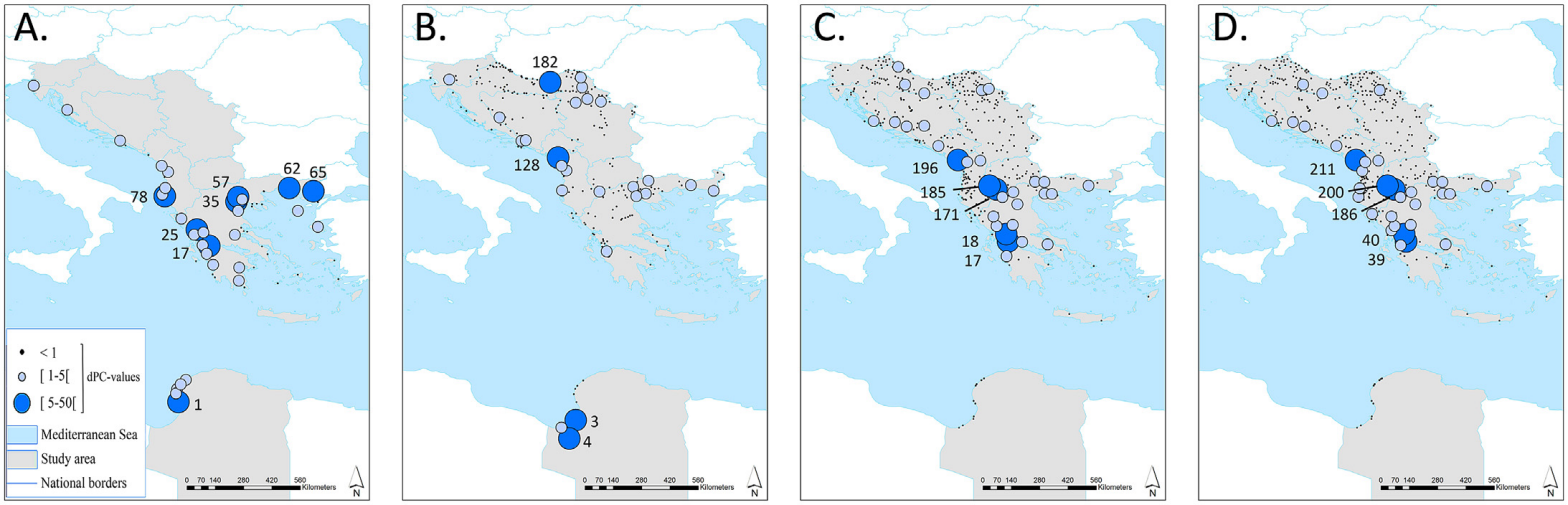
Visualisation of the node importance values (dPC) for Balkan-Cyrenaica for four different habitat groups (CLC2006). **A** coastal marshes, salinas and intertidal flats; **B** inland marshes; **C** water bodies; and **D** water bodies and coastal lagoons. Relative node importance values are indicated by centroids of which the size represents intervals of dPC values (see legend in bottom left corner). Important nodes with a dPC value between [5–50 [are indicated by node IDs on the map; for names and dPC values of all nodes with dPC> 1, see S1–S4 Tables. See [34] and [33] for datasets of national borders.

Libya and, within the Balkan, Greece appear to hold a considerable number of important nodes (dPC) to maintain the connectivity of the network, as summarised in Table 2 and Fig 4. In addition, the overall connectivity (ECA(PC)) values are intermediate to ECA_min_ and the total habitat area, hence we consider the network to be relatively well connected. In comparison to other Balkan countries included, nodes in Greece scored especially high dPC values and were therefore especially important for maintaining connectivity of the networks for the categories of the coastal marshes, salinas and intertidal flats and water bodies and coastal lagoons (Table 2). Due to the scarcity of permanent water bodies in Libya, there was a low importance of incorporated Libyan patches for this latter category. Scutari Lake (Albania-Montenegro) and Ohrid Lake (Albania-FYROM) were important nodes (high dPC values for the categories inland marshes, water bodies and coastal lagoons. In the case of the inland marshes, the Libyan Sebkha Al Kabirah and Sebkha Ajdabiya & Al Brayqah are the two dominant wetland units with the highest dPC values. This implies that some of the focal wetlands in our study are also of major importance for the connectivity at a larger scale. Furthermore, it is clear that the large majority of wetlands (>90% for inland marshes and inland (and coastal) water bodies; 58% for coastal marshes) has a dPC value lower than 1, indicating that comparatively they are of little importance for maintaining connectivity for the selected seven species. The extended node importance output (summarising all nodes with dPC> 1, including the name of the wetland) from Conefor is incorporated in the S1–S4 Tables.

### Wetland connectivity in Greece-Cyrenaica

For wetland habitats in Greece-Cyrenaica, the ECA(PC) values are intermediate to ECA_min_ and the total habitat area (Table 3). Therefore we can again conclude that the network is relatively well connected. As may be concluded from the overall connectivity results (ECA(PC)) there is only a minor decrease in the connectivity of wetlands, when considering reduction of available habitat by applying FID buffers (Table 3). Species with a broader spectrum of suitable habitat (eurytopic species), like *C*. *niger* or *E*. *garzetta*, generally have larger networks at their disposal than more specialised (stenotopic) species, like *A*. *purpurea*. The latter species, which according to the expert survey is also most impacted by disturbance in terms of reduced habitat availability, has values for the overall connectivity metrics several orders of magnitude smaller than the other species given its limited area of reachable habitat.

**Fig 5:**
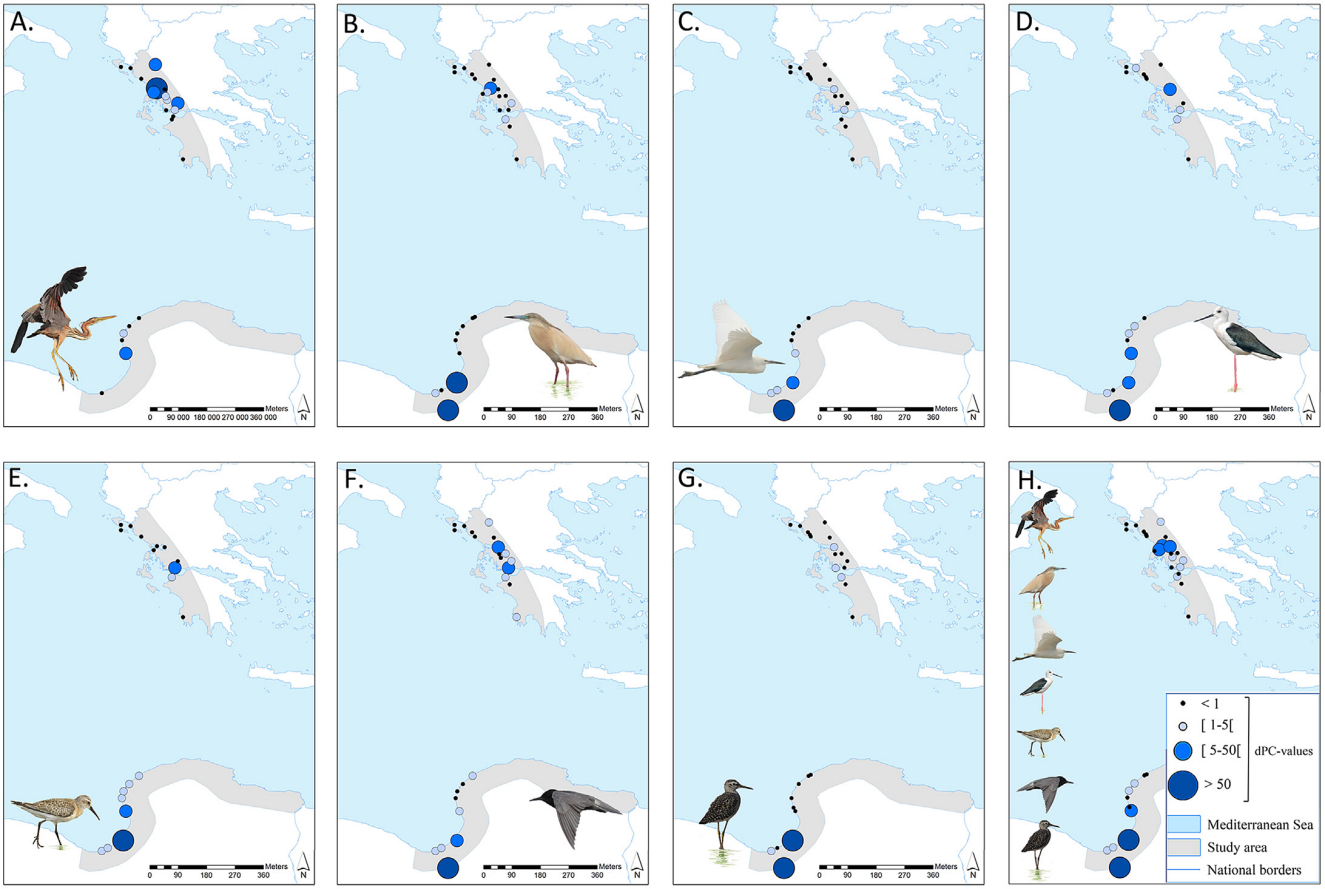
Visualisation of the node importance values (dPC) for Balkan-Cyrenaica for the total habitat maps. The size of the coloured centroids represents intervals of node importance values (dPC values; see legend in bottom right corner). The different maps indicate dPC values for: **A** *Ardea purpurea*; **B** *Ardeola ralloides*; **C** *Egretta garzetta*; **D** *Himantopus himantopus*; **E** *Calidris ferruginea*; **F** *Chlidonias niger*; **G** *Tringa glareola* and **H** the mean node importance values (dPC) for all wetland nodes, averaged for the number of species for which that node is present in the network. See [34] and [48] for datasets of national borders. Bird illustrations were provided by Joachim Teunen (A, B, C, F and G) and Joris Everaert (D and E).

Both in western Greece and eastern Libya, important connectivity sites can be identified (Fig 5). For all species except *A*. *purpurea*, the Libyan Sebkhas Al Kabirah (ID 1), Ajdabiya & Al Brayqah (ID 4), Sebkha Karkurah (ID 5) and Amvrakikos (ID 25) take a decisive position in the network. The reed beds of Amvrakikos (ID 27), Trichonida and Lysimacheia (ID 19), and Voulkaria (ID 24) are vital for the migratory connectivity of *A*. *purpurea* according to this analysis. In addition, larger wetlands complexes appeared to have higher node importance values (dPC), as a result of the high dPC*intra* and dPC*flux* fractions that make up the dPC metric and are especially influenced by the surface area of a node. The dPC*connector* fraction mostly equals zero, except for *C*. *niger* and, to a lesser extent, *H*. *himantopus* (i.e. all wetland complexes are equally important as stepping stones).

The summarised node importance values, which are mean dPC values for each node calculated based on the species-specific analyses, are represented in Fig 5H. There are some important similarities between these node importance patterns and the species-specific networks.

## Discussion

### Greek-Cyrenaican wetland connectivity

Migratory waterbirds rely on patchily distributed wetlands in a large unsuitable matrix to complete their life cycle. Protection of a single species requires multiple reserves distributed along the migration route [3]. Our study supports our initial assumption that wetlands of western Greece and north-east Libya (Cyrenaica) are very important to sustain a suitable habitat network for migratory waterbirds (cf. wetland selection criteria). Tackling this important migratory corridor at two different spatial extents we can point out the strategic geographical location of these wetlands near barriers such as the Sahara Desert and the Mediterranean Sea, upgrading them from (protected) ‘suitable areas’ to ‘most crucial stopovers’ which would require specific additional conservation and management efforts for these migratory waterbirds.

The connectivity of wetland habitats is evaluated through the use of the Equivalent Connected Area (ECA(PC)). Our ECA values are intermediate to ECA_min_ and the total habitat area, which correspond to the upper and lower range limits of ECA or to maximally and minimally connected networks respectively. Therefore we concluded the networks of wetland habitats at both extents, Balkan-Cyrenaica and Greece-Cyrenaica, to be relatively well connected (Table 3). As a result of the smaller amount of habitat suitable for stenotopic species such as *Ardea purpurea*, the overall connectivity (ECA(PC) value) of habitat networks is lower than for eurytopic species (e.g. *Chlidonias niger* and *Egretta garzetta*).

Migratory waterbirds appear to have more stepping stone wetlands at their disposal than absolutely necessary, as could be concluded from the low dPC*connector* fractions. This result is principally explained by the high interpatch dispersal probabilities of these highly mobile species, which were derived from the well-considered choice of the model’s input parameters: a maximum non-stop flight of 8,000 km, corresponding to a probability of 0.05. Therefore they might easily skip many relatively closely spaced stepping stones during migration. We conjectured to find strategically positioned but smaller wetlands such as Pylos (ID 12) to display high node importance values (dPC) but instead large wetland complexes recurrently appeared in the highest ranks of node importance values (dPC). High contributions of the fractions dPC*intra* and dPC*flux* are responsible for these results since these both take patch attribute (surface area) into account while dPC*connector* fraction contributions are of negligible importance.

### Balkan-Cyrenaica—a wider context

The Balkan-Cyrenaica analysis provided context of the surrounding wetland configuration of the nested fine-scale directed graph for Greece-Cyrenaica. It confirmed the importance of several Greek wetland complexes which scored especially high node importance values (dPC) in comparison to other included Balkan countries. It is however an analysis which lacks ground truthing data for the CORINE Land Cover (CLC2006) classifications and which is not explicitly species-specific. The incorporated habitat categories are however relevant as potential habitat for various types of migratory waterbirds.

Besides relying on different sources as classification baselines (GlobWetland II-classifications), wetland habitat availability in Libya is limited to a very narrow timeframe (‘pop-up supermarkets’; [49]), which might easily cause a mismatch in timing of migrants passing and habitat suitability. Since the absence of field validations generates an uncertainty of unidentifiable magnitude for the quality of Libyan habitat maps, we advise to be cautious with conclusions for this area. For all habitat maps it is important to note that within habitat patches, habitat quality and carrying capacity may still vary locally because of differences at the level of microhabitat features (benthic prey abundance, sediment type and size, etc.; e.g. [50, 51]). Additional factors such as more precise salinity maps, water depth, food availability could benefit the ecological relevance of our approach even further (e.g. [52]).

### Networks of key wetland sites

Our findings of the disproportionate importance of few larger wetland complexes for the ecological connectivity of migratory waterbirds corroborate the significance of conservation initiatives such as the Critical Site Network (CSN) [53] that specifically targets internationally important sites for waterbirds in the African-Eurasian Waterbird Agreement area (AEWA—developed under the UNEP's Convention on Migratory Species (Bonn Convention)). This initiative proposes to maintain a minimal functioning network of widely spaced critical wetland sites through complementary local, national and international conservation action is key to preserve healthy waterbird populations [54].

Most nodes appeared to have a low importance (low dPC value). It should be noted however that even if the individual loss of a given node (only one node removed at a time from the network) might not have a large effect on the connectivity metric value, the loss of all these “low-importance nodes” simultaneously from the network, might have a considerable effect on the connectivity of the network. The removal of individual patches from a network (one at a time) is a common approach to determine spatially-explicit priorities for conservation or to support land use change decisions, but it is now demonstrated—at least for species with low to average dispersal abilities—that certain combinations of removed patches and their interactions might be more detrimental for connectivity as could be expected from their individual node importance values [55].

In line with uncertainties of future environmental change, we also want to stress the importance of smaller wetlands as temporary emergency grounds allowing alternative routes when conditions in larger or recognised important wetlands have become unsuitable due to stochastic events or detrimental projects (local pollution, infrastructure works, extreme weather, etc.) [1, 22].

In order to understand the persistence of these populations, it is necessary to integrate a broad spectrum of environmental and physiological variables over the full migratory pathway. Our study focuses on a specific chain of wetlands as a section of flight routes across the eastern Mediterranean, and thereby contributes to a connection between actual network structure and waterbird conservation.

### Multi-species approach

By averaging ranks and dPC values of all focal species (Fig 5H; S5 Table), we obtained a ‘weighted’ importance of focal wetlands harbouring an ecological umbrella of species. For some species this ‘mean’ approach will prove to be more suitable (*i*.*e*. in accordance with the individual results) than for others (e.g. the specialist species *A*. *purpurea)*. Networks of protected areas must be designed and managed in order to account for the diverse suitability and connectivity needs of a comprehensive range of species. Therefore, it has been suggested to work with functional groups of species with comparable habitat requirements [12, 56]. This was exactly our approach by selecting common species with divergent habitats as representatives of such functional groups of a much wider diversity of waterbird species. Commonness is of course of practical concern in this study, because obtaining expert estimates of Flight Initiation Distances (FIDs) for rare species are less evident and possibly less reliable. Moreover, common species are disproportionately more important for ecosystem functioning and adaptation to environmental change.

### Human disturbance and Flight Initiation Distances

Human disturbance at the level of wetlands can have important implications for the habitat suitability and the connectivity of an entire habitat network. Especially during migration stopover, the accumulated effect (or impact at the population level) of small but frequent disturbance can have severe consequences [57, 58]. Higher energetic demands due to increased vigilance and escape flights, stress and reduced feeding time might reduce survival and breeding success [2, 41]. The results we obtained through our extensive expert survey are currently the most ecologically relevant and reliable quantitative expression of disturbance distances, particularly given a highly variable parameter such as FID, which depends on numerous variables (both for the sources of disturbance and the response of birds to these) [29]. We must emphasize that the active hunting tradition in the wider Mediterranean region will prevent habituation to human presence and might cause an even more intense reaction to human disturbance [59, 60]. We therefore assumed that roads, buildings and other types of man-made landscape structures (CLC 1.x.x., S2 File) are significant sources of human disturbance. Roads, enhancing ‘human connectivity’, have been shown to cause biodiversity loss, isolation of populations and increased mortality [61–63]. Our reasoning is that a best professional or expert estimate of an FID is better than assuming that there is no disturbance to birds by humans at all. Furthermore we acknowledge the relative small reduction of habitat area and more importantly, the transient impact of the assessed human disturbance, but we want to draw attention to this additional stress to migratory waterbirds and promote its inclusion when designing buffers. In practice, it is then possible to locate where controlling disturbance would be most effective for conservation.

### Climate change and habitat availability

Different components of global change such as climatic change and sea level rise are considered to exacerbate the degradation of Mediterranean wetlands, which are already suffering under high human pressure (e.g. [23]). For instance, sea level rise has the potential to significantly alter coastal wetlands as a result of inundation, erosion and salt water intrusion. Under a scenario of one meter sea level rise, Libya will suffer the highest loss (about 3,725 km^2^) of saline wetlands worldwide [64]. Moreover, based on a large-scale climatologic modelling approach of species distribution changes, Huntley et al. [65] concluded that as a result of climate change many breeding birds (including our focal species) in Europe will undergo rapid and large (~200 to 900 km) northward range shifts and contractions by the end of this century. Bellisario et al. [26] pointed out that climate impacts not only will change the distribution of suitable habitats for migrants, but also that most sites are unreliable in dealing with changing habitat conditions to ensure the long-term connectivity and persistence of species. When considering the climate projections for the Sahel and the Mediterranean region, we assume the distance between suitable wetlands north and south of the Palearctic-Afrotropical flyway’s major barriers will become even larger for migratory waterbirds. It is now the question in which way birds will to respond to this aggravated risk in their migratory journey, and which spatial configuration of stepping stones is necessary to allow adaptation.

## Conclusion

Greece and, to a lesser extent, Libya provide vital wetland sites upholding connectivity for migratory waterbirds when considered in a wider geographical context. Our results suggest that these wetlands are fairly well connected for these highly mobile migratory waterbird species and that few larger wetlands are of disproportionate importance for upholding their ecological connectivity. Since the current wetland configuration is internally well-connected at both geographic extents of this study, we argue that attention should be directed especially to wetland suitability and mitigation of disturbance in these stopover habitats. Except for the deep inland artificial lakes, all Greek wetland sites are protected but not necessarily designated under EU legislation for our focal species. Additionally, in practice, law enforcement and effective management for bird conservation is still insufficient. We advocate that additional and targeted management of few wetland complexes of the Natura 2000 Network could secure connectivity for waterbird populations, especially given limited conservation resources and projected environmental changes in the Mediterranean Region. Wetland management for bird conservation requires region-specific knowledge about waterbird communities and their seasonal dynamics [12] to allow for a better timing of appropriate management and ensure habitat suitability for different species in a wetland complex. We are aware that there is no single management solution for effective protection schemes of multiple taxa, given the various and sometimes even contrasting habitat requirements of different waterbirds and the requirement of setting priorities and trade-offs among different species [66, 67].

We do believe that the existing pragmatic approach of quantitative site selection criteria for conservation (e.g. Critical Site Network [53]) could be appended by integrating an as yet lacking functional connectivity component as established in this study. Functional connectivity of protected wetland areas should also be integrated into larger planning and management frameworks such as the African-Eurasian Migratory Waterbird Agreement (AEWA), the Ramsar Convention on Wetlands, the EU Birds Directive and the Bern Convention’s Emerald Network.

## Supporting Information

S1 File Expert survey form. (DOCX)

S2 File CORINE Land Cover codes for the wetland habitats used in the analysis. Adopted from [68]. (DOCX)

S1 Table Analysis of directed connectivity for Balkan-Cyrenaica (Libya) (dPC> 1) for coastal marshes. Nodes of coastal marshes (4.2.1.; 4.2.2.; 4.2.3.) with a dPC value larger than 1 are listed by descending dPC values. dA is the percentage of total habitat area. Locations in Greece and Libya are underlined, wetlands included at the spatial extent of Greece-Cyrenaica are in bold.
(DOCX)

S2 Table Analysis of directed connectivity for Balkan-Cyrenaica (Libya) (dPC> 1) for inland marshes. Nodes of inland marshes (4.1.1.) with a dPC value larger than 1 are listed by descending dPC values. dA is the percentage of total habitat area. Locations in Greece and Libya are underlined, wetlands included at the spatial extent of Greece-Cyrenaica are in bold.
(DOCX)

S3 Table Analysis of directed connectivity for Balkan-Cyrenaica (Libya) (dPC> 1) for inland water bodies. Nodes of water bodies (5.1.2.) with a dPC value larger than 1 are listed by descending dPC values. dA is the percentage of total habitat area. Locations in Greece and Libya are underlined, wetlands included at the spatial extent of Greece-Cyrenaica are in bold.
(DOCX)

S4 Table Analysis of directed connectivity for Balkan-Cyrenaica (Libya) (dPC> 1) for inland and coastal water bodies. Nodes of water bodies (5.1.2.; 5.2.1.) with a dPC value larger than 1 are listed by descending dPC values. dA is the percentage of total habitat area. Locations in Greece and Libya are underlined, wetlands included at the spatial extent of Greece-Cyrenaica are in bold.
(DOCX)

S5 Table Analysis of directed connectivity for Greece-Cyrenaica: node importance (dPC) values of focal wetlands for each species-specific analysis. The mean dPC value for each node is calculated by dividing the summed dPC value for each node by the number of species for which that node is part of the network (non-empty cells).
(DOCX)
