# Supplementary material for: Wetland Suitability and Connectivity for Trans-Saharan Migratory Waterbirds
Source: PLoS One. 2015 Aug 10;10(8):e0135445. doi: 10.1371/journal.pone.0135445 (PMC4530951; doi:10.1371/journal.pone.0135445)
Supplement: S1 File — (DOCX) [file pone.0135445.s001.docx]

**File S1: Expert survey form**

**Human disturbance distances to waterbirds during migration/stopover**

**Background:**

Animals’ avoidance of humans or human activities can have several adverse effects on their distribution and abundance. Human disturbance might even be to blame for declines of waterbirds because of the frequency of occurrence can lead to decreased foraging time, and might lead to a reduced area of suitable foraging or roosting habitat. A frequent tool used by conservation managers to avoid such effects is to designate ‘buffer zones’ (or set-back distances or protection zones) around centres of animals’ distribution within which human activity is restricted. Assigning buffers is almost exclusively done by the use of **expert-knowledge** since there is **no empirical basis** for specifying any particular buffer width for every disturbance type to every bird species. Our reasoning/standpoint is that a best professional ‘guess’ for a buffer width is better than assuming that there is no human disturbance to bird behaviour at all.

**Goal:**

We want to ‘guesstimate’ (an estimate that combines reasoning with guessing) two different types of disturbances (‘static’ and ‘active’) to a selection of nine waterbird species during stopover periods. Therefore we call on you as an ‘expert’ to give your best professional judgement based on the autoecology and known behaviour of the different bird species to assign these buffers in a conservative  way (conservative = so no irreal large buffers).

**Question**:

We would like to ask you to fill in the table on the next page (grey columns only) if you are capable of doing so/if you have field experience with some (or all) of these species. A second, more detailed table is inserted in case if you have more specific knowledge on one (or more) certain species. You can always forward it to other knowledgeable ornithologists who might be willing to share their experience and knowledge with us. You can always reach us through email (see below).

**Further information: ‘Static’ and ‘active’ disturbance distances**

(from: Ruddock & Whitfield, 2007. A review of disturbance distances in selected bird species)

A common method used to prescribe buffer zones involves one or two measures of disturbance distance: ‘**alert distance**’ (AD), the distance between the disturbance source and the animal at the point where the animal changes its behaviour in response to the approaching disturbance source, and ‘**flight initiation distance**’ (FID), the point at which the animal flushes or otherwise moves away from the approaching disturbance source.

Thank you.

Ronny Merken Evelien Deboelpaep

PhD student MSc student

rmerken@vub.ac.be evdeboel@vub.ac.be

Table 1 - Expert survey form: Data overview of bird species selection (Burfield & Van Bommel, 2004, Cramp, 2004, Delany *et al.*, 2009, Handrinos & Akriotis, 1997, Mullarney *et al.*, 2010, Shirihai & Christie, 1996)

| **SPECIES** | | **HABITAT** | **STATUS** | | **MIGRATION** | | **BIRDLIFE STATUS** | **IUCN RED LIST** | **NATURA 2000** | **DISTURBANCE DISTANCE^[[1]](#footnote-1)^ (m)** | |
| --- | --- | --- | --- | --- | --- | --- | --- | --- | --- | --- | --- |
| ***Scientific name*** | **English name** |  | **EU** | **Greece** | **Period** | **Peak** |  |  | **ANNEX I** | **Alert distance (AD)** | **Flight initiation distance**  **(FID)** |
| *Calidris ferruginea* | Curlew sandpiper | Hypersaline habitats with sparse vegetation - saltpans, coastal lagoons & *Arthrocnemum*-dominated saltmarshes | Non-breeding | Only passage, numbers unknown | 15/04 - 10/06 | 25/04-15/05 | NE | LC | N |  |  |
| *Plegadis falcinellus* | Glossy ibis | Freshwater or brackish wetlands; wet meadows, lake margins, coastal lagoons | < 22.000 | 150-200 | 25/03-15/05 | 01/04-15/04 | D-MCD | LC | Y |  |  |
| *Tringa glareola* | Wood sandpiper | Well vegetated fresh or brackish marshland | >350.000 | Only passage, numbers unknown | 25/03-15/05 | 15-31/04 | D-MHD | LC | Y |  |  |
| *Ardea purpurea* | Purple heron | Reedbeds (mainly freshwater) | < 42.000 | 105-150 | 25/03-10/05 | 10-20/04 | D-MCD | LC | Y |  |  |
| *Egretta garzetta* | Little egret | Large ecological amplitude - fresh, brackish and saline environments | < 94.000 | 1500-1800 | 15/03-25/05 | 15/04-10/05 | S | LC | Y |  |  |
| *Ardeola ralloides* | Squacco heron | Freshwater wetlands with abundant vegetation, likes canals and ditches | < 27.000 | 400 -700 | 25/03-25/05 | 25/04-10/05 | D-MCD | LC | Y |  |  |
| *Charadrius alexandrinus* | Kentish plover | Sea-coasts & mudflats | <35.000 | 1000-2000 | 10/03-10/05 | 15-31/04 | D-MCD | LC | Y |  |  |
| *Himantopus himantopus* | Black-winged stilt | Shores of large inland water bodies and estuarine or coastal habitats such as river deltas, coastal lagoons, and shallow freshwater or brackish pools with extensive areas of mudflats, salt meadows, saltpans, coastal marshes and swamps | < 64.000 | 1000-3000 | 25/03-15/05 | 15/04-10/05 | S | LC | Y |  |  |
| *Glareola pratincola* | Collared pratincole | Saltpans, riversides, lagoons & farmland | <18.000 | 500-1000 | 10/04-15/05 | 15-31/04 | D-MCD | LC | Y |  |  |

*NE: Not evaluated, D-MCD: declining - moderate continuing decline, D-MHD: depleted - moderate historical decline, S: secure, LC: Least Concern, Y: Yes, N: No*

Table 2: Expert survey form: Detailed table (indicate species and, if possible, type of environment) – You can copy this table if you wish to enter guesstimates for more than one species.

| **Species:**  ....................................  **Type of environment:**  *.....................................* | | | **Vehicle and direction of movement**  **Disturbance distance (m) (AD & FID^^[[2]](#footnote-2)^^)** | | | | | | | | | | | | | | | | | | | |  |
| --- | --- | --- | --- | --- | --- | --- | --- | --- | --- | --- | --- | --- | --- | --- | --- | --- | --- | --- | --- | --- | --- | --- | --- |
|  |  |  | On foot | | | | | Bicycle | | | | | Car | | | | | Boat | | | | |  |
|  |  |  | *Towards bird* | | *Passing* | |  | | *Towards bird* | | *Passing* | | | *Towards bird* | | *Passing* | | | *Towards bird* | | *Passing* | | |
|  |  |  | **AD** | **FID** | **AD** | **FID** |  | | **AD** | **FID** | **AD** | **FID** | | **AD** | **FID** | **AD** | **FID** | | **AD** | **FID** | **AD** | **FID** | |
| **Activity & number of birds** | Roosting | One/few bird(s) |  |  |  |  |  | |  |  |  |  | |  |  |  |  | |  |  |  |  | |
|  |  | Group of birds |  |  |  |  |  | |  |  |  |  | |  |  |  |  | |  |  |  |  | |
|  | Foraging | One/few bird(s) |  |  |  |  |  | |  |  |  |  | |  |  |  |  | |  |  |  |  | |
|  |  | Group of birds |  |  |  |  |  | |  |  |  |  | |  |  |  |  | |  |  |  |  | |

1. See former page for definitions of the terms [↑](#footnote-ref-1)
2. See first page for definitions of the terms [↑](#footnote-ref-2)
