# Supplementary material for: Wetland Suitability and Connectivity for Trans-Saharan Migratory Waterbirds
Source: PLoS One. 2015 Aug 10;10(8):e0135445. doi: 10.1371/journal.pone.0135445 (PMC4530951; doi:10.1371/journal.pone.0135445)
Supplement: S2 File — (DOCX) [file pone.0135445.s002.docx]

**S2 File. CORINE Land Cover codes for the wetland habitats used in the analysis**. Adopted from [[73](#_ENREF_70)].

1. **Artificial areas**
   1. Urban fabric
      1. Continuous urban fabric Surfaces covered for more than 80% by buildings and transport networks
      2. Discontinuous urban fabric Surfaces covered for 30-80% by buildings and transport networks
   2. Industrial, commercial and transport units
      1. Industrial or commercial units Surfaces predominated by artificially surfaced areas, which also contain buildings and/or vegetation
      2. Road and rail networks Motorways and railways, including associated installations
      3. Port areas Infrastructure of ports (quays, dockyards and marinas)
      4. Airports Airport installations (runways, buildings and associated land)
   3. Mine, dump and construction sites
      1. Mineral extraction sites Areas with open-pit extraction of construction material or other minerals
      2. Dump sites Public, industrial or mine dump sites
      3. Construction sites Spaces under construction development, soil or bedrock excavations, earthworks
   4. Artificial non-agricultural vegetated areas
      1. Green urban areas Areas with vegetation with urban fabric (parks, mansions, cemeteries etc.)
      2. Sport and leisure facilities Camping and sports grounds, leisure parks etc.
2. **Agricultural areas**
   1. Heterogeneous agricultural areas

2.4.3. Land principally occupied by agriculture, with significant areas of natural vegetation

1. **Forests and semi-natural areas**
   1. Open spaces with little or no vegetation
      1. Beaches, dunes and sand plains Beaches, dunes and expanses of sand or pebbles in coastal or continental locations, including beds of

stream channels with torrential regime

1. **Wetlands**
   1. Inland wetlands
      1. Inland marshes Low-lying land usually flooded in winter, and more or less saturated by water all year round
   2. Coastal wetlands
      1. Salt marshes Vegetated low-lying areas, above the high-tide line, susceptible to flooding by seawater. Often in the process of filling

in, gradually being colonized by halophilic plants

- - 1. Salines Salt-pans, active or in process of abandonment. Sections of salt marsh exploited for the production of salt by

evaporation. They are clearly distinguishable from the rest of the marsh by their parcellation and embankment systems

- - 1. Intertidal flats Generally unvegetated expanses of mud, sand or rock lying between high and low water marks

1. **Water bodies**
   1. Inland waters
      1. Water courses Natural or artificial water-courses serving as water drainage channels. Includes canals
      2. Water bodies Natural or artificial stretches of water.
   2. Coastal waters
      1. Coastal lagoons Stretches of salt or brackish water in coastal areas which are separated from the sea by a tongue of land or other

similar topography. These water bodies can be temporarily or permanently connected to the sea at limited points.

- - 1. Estuaries The mouth of a river within which the tide ebbs and flows
