# Supplementary material for: Wetland Suitability and Connectivity for Trans-Saharan Migratory Waterbirds
Source: PLoS One. 2015 Aug 10;10(8):e0135445. doi: 10.1371/journal.pone.0135445 (PMC4530951; doi:10.1371/journal.pone.0135445)
Supplement: S1 Table — Nodes of coastal marshes (4.2.1.; 4.2.2.; 4.2.3.) with a dPC value larger than 1 are listed by descending dPC values. dA is the percentage of total habitat area. Locations in Greece and Libya are underlined, wetlands included at the spatial extent of Greece-Cyrenaica are in bold. (DOCX) [file pone.0135445.s003.docx]

**S1 Table – Analysis of directed connectivity for Balkan-Cyrenaica (Libya) (dPC> 1) for coastal marshes.** Nodes of coastal marshes (4.2.1.; 4.2.2.; 4.2.3.) with a dPC value larger than 1 are listed by descending dPC values. dA is the percentage of total habitat area. Locations in Greece and Libya are underlined, wetlands included at the spatial extent of Greece-Cyrenaica are in bold.

| **Node** | **dA** | **dPC** | **Location** | **Countries** |
| --- | --- | --- | --- | --- |
| 62 | 13.74668 | 26.1516 | Ethniko Parko Anatolikis Makedonias kai Thrakis (Nestou Delta) | Greece |
| 25 | 11.50033 | 22.2513 | **Amvrakikos (north)** | Greece |
| 65 | 11.14513 | 20.7988 | Evros Delta | Greece |
| 57 | 7.405017 | 14.3589 | Axios & Aliakmonas Delta | Greece |
| 1 | 8.735612 | 14.0277 | **Sebkha Karkurah** | Libya |
| 17 | 7.064469 | 13.6285 | **Messolonghi (east)** | Greece |
| 35 | 2.799949 | 5.44002 | Kitros Salines | Greece |
| 78 | 2.874763 | 5.36232 | Laguna e Nartës | Albania |
| 79 | 2.646128 | 4.78921 | Ulcinjska Saline | Montenegro |
| 48 | 2.235442 | 4.14752 | Parku Kombëtar Divjakë-Karavasta | Albania |
| 5 | 2.51907 | 4.14205 | **Sebkha El Thama/Esselawi** | Libya |
| 2 | 2.18171 | 3.56320 | **Sebkha Gandoufa** | Libya |
| 34 | 1.625769 | 3.16381 | Kastri-Neoi Poroi | Greece |
| 63 | 1.563611 | 2.95541 | Katafigio Agrias Zois Komi-Tourles-Chortalimni | Greece |
| 30 | 1.412286 | 2.73514 | Sperchiou Delta | Greece |
| 16 | 1.381029 | 2.65879 | **Messolonghi (west)** | Greece |
| 36 | 1.17989 | 2.17012 | Kallonis Gulf | Greece |
| 56 | 1.088624 | 2.11266 | Aggelochori Salines - Epanomis - Tsairi | Greece |
| 26 | 0.9266428 | 1.76852 | **Kalamas** | Greece |
| 4 | 0.9782163 | 1.58825 | **Ghemines** | Libya |
| 50 | 0.8534226 | 1.56325 | Këneta e Vainit-Kunis, Rezervati Kune-Vain-Tale | Albania |
| 46 | 0.8231317 | 1.53017 | Lumi Vjosa River (estuary) | Albania |
| 74 | 0.9545464 | 1.38011 | Sečoveljske Saline | Slovenia-Croatia |
| 18 | 0.7143261 | 1.36721 | **Kotychi** | Greece |
| 42 | 0.8714324 | 1.34432 | Velo I Malo Blato | Croatia |
| 24 | 0.6153644 | 1.19233 | **Amvrakikos (east)** | Greece |
| 11 | 0.6377024 | 1.18346 | Eurotas Delta | Greece |
| 21 | 0.6068407 | 1.16977 | **Lefkada-Voulkaria-Saltini** | Greece |
| 9 | 0.5833238 | 1.10531 | Kaiafas Lake | Greece |
| 39 | 0.6206028 | 1.04791 | Neretva Delta | Croatia |
| 12 | 0.5342424 | 1.00583 | Ygroviotopos Moustou | Greece |
| 3 | 0.6064217 | 1.00254 | **Sebkha Al Kuz** | Libya |
