# Supplementary material for: Wetland Suitability and Connectivity for Trans-Saharan Migratory Waterbirds
Source: PLoS One. 2015 Aug 10;10(8):e0135445. doi: 10.1371/journal.pone.0135445 (PMC4530951; doi:10.1371/journal.pone.0135445)
Supplement: S2 Table — Nodes of inland marshes (4.1.1.) with a dPC value larger than 1 are listed by descending dPC values. dA is the percentage of total habitat area. Locations in Greece and Libya are underlined, wetlands included at the spatial extent of Greece-Cyrenaica are in bold. (DOCX) [file pone.0135445.s004.docx]

**S2 Table – Analysis of directed connectivity for Balkan-Cyrenaica (Libya) (dPC> 1) for inland marshes.**

Nodes of inland marshes (4.1.1.) with a dPC value larger than 1 are listed by descending dPC values. dA is the percentage of total habitat area. Locations in Greece and Libya are underlined, wetlands included at the spatial extent of Greece-Cyrenaica are in bold.

| **Node** | **dA** | **dPC** | **Location** | **Countries** |
| --- | --- | --- | --- | --- |
| 4 | 26.75897 | 47.256 | **Sebkha Al Kabirah** | Libya |
| 3 | 17.74849 | 31.6545 | **Sebkha Ajdabiya & Al Brayqah** | Libya |
| 128 | 6.406291 | 11.2488 | Scutari Lake | Montenegro-Albania |
| 182 | 6.759103 | 11.1567 | Duna-Dráva Nemzeti Park (Duna River) | Serbia-Croatia |
| 213 | 2.672056 | 4.44837 | Tisa River (central) | Serbia |
| 48 | 2.10096 | 3.59886 | Neretva Delta | Bosnia and Herzegovina - Croatia |
| 157 | 1.918233 | 3.16 | Evros Delta | Greece |
| 224 | 1.422954 | 2.39345 | Danube River (east) | Serbia |
| 215 | 1.452744 | 2.38662 | Tisa River (north) | Serbia |
| 142 | 1.317532 | 2.28164 | Kerkini Lake | Greece |
| 159 | 1.252173 | 2.10372 | Vistonida Lake | Greece |
| 115 | 1.076705 | 1.90382 | Prespa Lake | Greece-Albania-Macedonia |
| 253 | 1.05524 | 1.81138 | Neretva Delta | Croatia-Bosnia and Herzegovina |
| 111 | 1.004674 | 1.76888 | Bojana-Buna Delta | Albania- Montenegro |
| 69 | 1.135285 | 1.73571 | Notranski Regijski Park | Slovenia |
| 192 | 0.9437691 | 1.58961 | Danube River - Tisa (central) | Serbia |
| 61 | 0.9538814 | 1.5833 | Kazanci Region | Bosnia and Herzegovina |
| 105 | 0.882108 | 1.5554 | Këneta e Vainit-Kunis, Rezervati Kune-Vain-Tale | Albania |
| 106 | 0.7883119 | 1.39221 | Parku Kombëtar Divjakë-Karavasta | Albania |
| 139 | 0.6993326 | 1.21669 | Koroneia Lake | Greece |
| 17 | 0.6511428 | 1.14646 | **Strofilia-Araxos** | Greece |
| 1 | 0.6327135 | 1.11943 | **Sebkha Bishr** | Libya |
| 145 | 0.6340611 | 1.10905 | Axios Delta | Greece |
| 127 | 0.6308001 | 1.10312 | Axios River (north) | Greece |
| 185 | 0.6241496 | 1.05619 | Special Nature Reserve Obedska Bara, Sava River | Serbia |
