# Supplementary material for: Wetland Suitability and Connectivity for Trans-Saharan Migratory Waterbirds
Source: PLoS One. 2015 Aug 10;10(8):e0135445. doi: 10.1371/journal.pone.0135445 (PMC4530951; doi:10.1371/journal.pone.0135445)
Supplement: S3 Table — Nodes of water bodies (5.1.2.) with a dPC value larger than 1 are listed by descending dPC values. dA is the percentage of total habitat area. Locations in Greece and Libya are underlined, wetlands included at the spatial extent of Greece-Cyrenaica are in bold. (DOCX) [file pone.0135445.s005.docx]

**S3 Table – Analysis of directed connectivity for Balkan-Cyrenaica (Libya) (dPC> 1) for inland water bodies**. Nodes of water bodies (5.1.2.) with a dPC value larger than 1 are listed by descending dPC values. dA is the percentage of total habitat area. Locations in Greece and Libya are underlined, wetlands included at the spatial extent of Greece-Cyrenaica are in bold.

| **Node** | **dA** | **dPC** | **Location** | **Countries** |
| --- | --- | --- | --- | --- |
| 196 | 13.07845 | 25.5524 | Scutari Lake | Albania-Montenegro |
| 185 | 12.64879 | 24.9839 | Ohrid Lake | Albania-Macedonia |
| 171 | 10.76195 | 21.2120 | Prespa Lakes | Albania-Macedonia-Greece |
| 18 | 3.680538 | 6.89083 | **Techniti Limni Kremaston & Kastrakiou-Stratiou** | Greece |
| 17 | 3.69579 | 6.84077 | **Lysimacheia & Trichonis Lakes** | Greece |
| 228 | 2.411989 | 4.53326 | Volvi Lake | Greece |
| 380 | 2.269672 | 4.37364 | Techniti Limni Polifitou | Greece |
| 231 | 2.297215 | 4.36956 | Kerkini Lake | Greece |
| 181 | 1.788359 | 3.48580 | Petron & Vegoritida Lakes | Greece |
| 216 | 1.732007 | 3.39785 | Fierzes Reservoir Lake - White Drin River | Albania-Kosovo(Serbia) |
| 62 | 1.739336 | 3.20300 | Buško Lake | Bosnia and Herzegovina |
| 211 | 1.543701 | 2.77846 | Vistonida Lake/Lagoon | Greece |
| 230 | 1.227863 | 2.35679 | Doiran Lake | Greece-Macedonia |
| 194 | 1.164928 | 2.28324 | Liqeni Vau e Dejës | Albania |
| 39 | 1.114682 | 1.99656 | Iliki & Paralimni Lakes | Greece |
| 178 | 1.021453 | 1.99600 | Orestiada Lake | Greece |
| 56 | 1.07991 | 1.91495 | Vransko Lake | Croatia |
| 119 | 0.8667377 | 1.56664 | Jelaš Ribnjaci-dio | Croatia |
| 198 | 0.7744612 | 1.48065 | Bilećko Lake | Bosnia and Herzegovina |
| 227 | 0.7758131 | 1.46966 | Koroneia Lake | Greece |
| 28 | 0.7625491 | 1.44063 | Techniti Limni Plastira | Greece |
| 29 | 0.7077557 | 1.35771 | **Ioannina Lake** | Greece |
| 123 | 0.7340481 | 1.29531 | Ribnjaci Lakes | Croatia |
| 277 | 0.7081309 | 1.29327 | Belo Lakes - Tisa River | Serbia |
| 26 | 0.6217844 | 1.17849 | **Techniti Limni Pournariou** | Greece |
| 66 | 0.6390281 | 1.16027 | Peručko Lake | Croatia |
| 40 | 0.5872489 | 1.07760 | Techniti Limni Mournou | Greece |
| 362 | 0.6074807 | 1.04038 | Dubravsko Lake | Croatia |
| 13 | 0.5706096 | 1.03008 | **Techniti Limni Pineiou** | Greece |
| 382 | 0.5488965 | 1.02491 | Jablaničko Lake | Bosnia and Herzegovina |
| 307 | 0.5626846 | 1.02480 | Ribnjac Lakes - Tamis River | Serbia |
