# Supplementary material for: Wetland Suitability and Connectivity for Trans-Saharan Migratory Waterbirds
Source: PLoS One. 2015 Aug 10;10(8):e0135445. doi: 10.1371/journal.pone.0135445 (PMC4530951; doi:10.1371/journal.pone.0135445)
Supplement: S4 Table — Nodes of water bodies (5.1.2.; 5.2.1.) with a dPC value larger than 1 are listed by descending dPC values. dA is the percentage of total habitat area. Locations in Greece and Libya are underlined, wetlands included at the spatial extent of Greece-Cyrenaica are in bold. (DOCX) [file pone.0135445.s006.docx]

**S4 Table – Analysis of directed connectivity for Balkan-Cyrenaica (Libya) (dPC> 1) for inland and coastal water bodies**. Nodes of water bodies (5.1.2.; 5.2.1.) with a dPC value larger than 1 are listed by descending dPC values. dA is the percentage of total habitat area. Locations in Greece and Libya are underlined, wetlands included at the spatial extent of Greece-Cyrenaica are in bold.

| **Node** | **dA** | **dPC** | **Location** | **Countries** |
| --- | --- | --- | --- | --- |
| 211 | 11.7069 | 22.9692 | Scutari Lake | Albania-Montenegro |
| 200 | 11.3223 | 22.5236 | Ohrid Lake | Albania-Macedonia |
| 186 | 9.633331 | 19.1301 | Prespa Lakes | Albania-Macedonia-Greece |
| 40 | 3.294556 | 6.25987 | **Techniti Limni Kremaston & Kastrakiou-Stratiou** | Greece |
| 39 | 3.308209 | 6.21911 | **Lysimacheia & Trichonis Lakes** | Greece |
| 7 | 2.208034 | 4.22483 | **Amvrakikos (north)** | Greece |
| 242 | 2.159041 | 4.09439 | Volvi Lake | Greece |
| 393 | 2.031649 | 3.95417 | Techniti Limni Polifitou | Greece |
| 245 | 2.056303 | 3.94062 | Kerkini Lake | Greece |
| 246 | 1.992501 | 3.61665 | Vistonida Lagoon | Greece |
| 10 | 1.818783 | 3.40319 | **Messolonghi** | Greece |
| 21 | 1.72349 | 3.38760 | Parku Kombëtar Divjakë-Karavasta | Albania |
| 196 | 1.600812 | 3.14563 | Petron & Vegoritida Lakes | Greece |
| 230 | 1.550369 | 3.05531 | Fierzes Reservoir Lake - White Drin River | Albania-Kosovo(Serbia) |
| 81 | 1.55693 | 2.86954 | Buško Lake | Bosnia and Herzegovina |
| 244 | 1.099095 | 2.12494 | Doiran Lake | Greece-Macedonia |
| 209 | 1.042761 | 2.05333 | Liqeni Vau e Dejës | Albania |
| 5 | 0.9928782 | 1.94169 | Laguna e Nartës | Albania |
| 59 | 0.997784 | 1.81305 | Iliki & Paralimni Lakes | Greece |
| 193 | 0.9143326 | 1.80261 | Orestiada lake | Greece |
| 75 | 0.9666586 | 1.71508 | Vransko Lake | Croatia |
| 139 | 0.775842 | 1.40047 | Jelaš Ribnjaci - dio | Croatia |
| 213 | 0.6932426 | 1.32922 | Bilećko Lake | Bosnia and Herzegovina |
| 241 | 0.6944528 | 1.32715 | Pivsko Lake | Montenegro |
| 48 | 0.6825798 | 1.30681 | Techniti Limni Plastira | Greece |
| 49 | 0.6335326 | 1.23060 | **Ioannina Lake** | Greece |
| 14 | 0.6230773 | 1.20983 | **Kalamas** | Greece |
| 143 | 0.6570677 | 1.15743 | Ribnjaci Lakes | Croatia |
| 290 | 0.6338685 | 1.15684 | Belo Lakes - Tisa River | Serbia |
| 46 | 0.5565772 | 1.06990 | **Techniti Limni Pournariou** | Greece |
| 85 | 0.5720126 | 1.03913 | Peručko Lake | Croatia |
| 22 | 0.5269399 | 1.03893 | Këneta e Vainit-Kunis, Rezervati Kune-Vain-Tale | Albania |
