# Supplementary material for: Wetland Suitability and Connectivity for Trans-Saharan Migratory Waterbirds
Source: PLoS One. 2015 Aug 10;10(8):e0135445. doi: 10.1371/journal.pone.0135445 (PMC4530951; doi:10.1371/journal.pone.0135445)
Supplement: S5 Table — The mean dPC value for each node is calculated by dividing the summed dPC value for each node by the number of species for which that node is part of the network (non-empty cells). (DOCX) [file pone.0135445.s007.docx]

**S5 Table**: Analysis of directed connectivity for Greece-Cyrenaica: node importance (dPC) values of focal wetlands for each species-specific analysis. The mean dPC value for each node is calculated by dividing the summed dPC value for each node by the number of species for which that node is part of the network (non-empty cells).

| **Node** | **Wetland** | ***Ardea purpurea*** | ***Ardeola ralloides*** | ***Calidris ferruginea*** | ***Chlidonias niger*** | ***Egretta garzetta*** | ***Himantopus himantopus*** | ***Tringa glareola*** | **Mean** |
| --- | --- | --- | --- | --- | --- | --- | --- | --- | --- |
| 1 | Sebkha Al Kabirah |  | 75.17677 |  | 61.62578 | 75.32367 | 71.74999 | 75.98095 | 71.97143 |
| 2 | Sekbha Bishr | 0.04868 | 1.76265 | 3.47469 | 1.52402 | 1.82776 | 1.68203 | 1.77607 | 1.72798 |
| 3 | Sebkha Hafiroun & Brega |  | 0.84000 | 4.03295 | 1.59456 | 1.68615 | 0.80378 | 0.84845 | 1.63432 |
| 4 | Sebkha Ajdabiya & Al Brayqah |  | 49.70102 | 90.88125 | 40.18471 | 48.69121 | 47.59971 | 50.19165 | 54.54159 |
| 5 | Sebkha Karkurah (South) | 6.12967 | 0.51311 | 14.16718 | 4.16322 | 4.75857 | 6.35852 | 0.00133 | 5.15594 |
| 6 | Sebkha Karkurah (North) |  |  |  |  |  |  | 0.00256 | 0.00256 |
| 7 | Ghemines | 0.43174 | 0.03500 | 2.23695 | 0.64103 | 0.74900 | 0.82668 | 0.00001 | 0.70292 |
| 8 | Sebkha Gandoufa | 2.07425 | 0.16535 | 2.48707 | 0.75238 | 0.83150 | 1.74852 |  | 1.34318 |
| 9 | Sebkha El Thama & Esselawi | 0.83596 | 0.06721 | 2.75052 | 0.90971 | 0.91859 | 2.10272 | 0.00124 | 1.08371 |
| 10 | Sebkha Al Kuz (South) | 0.05716 | 0.00446 | 1.67206 | 1.38623 | 0.55874 | 0.50569 | 0.00002 | 0.59776 |
| 11 | Sebkha Al Kuz (North) |  | 0.00001 |  |  |  |  | 0.00001 | 0.00001 |
| 12 | Pylos | 0.96644 | 0.09810 | 0.08063 | 1.02003 | 0.22770 | 0.13019 | 0.12371 | 0.37811 |
| 13 | Techniti Limni Pineiou |  | 0.07573 |  | 0.74916 | 0.02645 |  | 0.08156 | 0.23323 |
| 15 | Strofilia, Kotychi & Araxos (South) | 0.31752 | 1.36814 | 1.27794 | 2.14390 | 0.88289 | 1.88512 | 1.68283 | 1.36548 |
| 16 | Strofilia, Kotychi & Araxos (North | 0.11090 |  |  |  |  |  |  | 0.11090 |
| 17 | Messolonghi (South) | 1.61476 | 0.02947 | 6.83718 | 5.13589 | 2.46896 | 4.37553 | 0.11847 | 2.94004 |
| 18 | Messolonghi (North) | 0.77041 | 0.76040 |  |  |  |  | 1.22468 | 0.91850 |
| 19 | Trichonis & Lysimacheia | 18.46912 | 0.91985 | 0.00075 | 4.83780 | 0.00236 | 0.00492 | 0.00821 | 3.46329 |
| 20 | Ozeros | 3.06722 |  |  | 0.43875 |  |  |  | 1.75299 |
| 21 | Techniti Limni Kastrakiou-Stratiou |  | 0.00843 |  | 1.35387 | 0.00173 |  | 0.00580 | 0.34246 |
| 22 | Amvrakia | 1.07471 | 0.32086 |  | 0.55641 | 0.06964 |  | 0.64400 | 0.53312 |
| 23 | Lefkada |  | 0.14096 |  |  |  |  |  | 0.14096 |
| 24 | Voulkaria | 17.45213 | 1.15999 | 0.52692 |  |  |  |  | 6.37968 |
| 25 | Amvrakikos (South) | 0.85812 | 0.04210 | 0.03508 | 25.84267 | 2.16632 | 5.40990 | 3.93300 | 5.46960 |
| 26 | Amvrakikos (East) | 0.11496 | 0.00089 | 0.00663 |  |  |  | 0.02581 | 0.03707 |
| 27 | Amvrakikos (West) | 84.69374 | 5.46352 | 0.38799 |  |  |  |  | 30.18175 |
| 28 | Amvrakikos (North) |  |  | 3.58119 |  |  |  |  | 3.58119 |
| 29 | Techniti Limni Pournariou |  | 0.00321 |  | 0.71852 | 0.09352 |  | 0.00294 | 0.20455 |
| 30 | Acheron | 0.31055 | 0.75507 | 0.01678 | 0.35054 | 0.24709 | 0.64680 | 0.81974 | 0.44951 |
| 31 | Kalodiki (South) |  |  |  |  | 0.00849 | 0.02248 | 0.00471 | 0.01189 |
| 32 | Kalodiki (North) |  | 0.07159 |  | 0.02804 | 0.02408 | 0.06380 | 0.06543 | 0.05059 |
| 33 | Korissia |  | 0.00003 | 0.06060 | 0.19100 | 0.02053 | 0.02785 |  | 0.06000 |
| 34 | Kalamas | 0.03267 | 0.45202 | 0.93204 | 0.67711 | 0.48173 | 1.26095 | 0.85294 | 0.66992 |
| 35 | Chalikiopoulou | 0.53249 | 0.03980 | 0.02861 | 0.08441 | 0.01445 | 0.03555 | 0.02492 | 0.10860 |
| 36 | Ioannina | 5.39304 | 0.30510 |  | 0.99348 | 0.01412 | 0.03670 | 0.03804 | 1.13008 |
